# New Fast Fall Detection Method Based on Spatio-Temporal Context Tracking of Head by Using Depth Images

**DOI:** 10.3390/s150923004

**Published:** 2015-09-11

**Authors:** Lei Yang, Yanyun Ren, Huosheng Hu, Bo Tian

**Affiliations:** 1 School of Mechatronic Engineering and Automation, Shanghai University, Shanghai 200072, China; E-Mails: yangyoungya@sina.com (L.Y.); ryypine@163.com (Y.R.); 2 School of Computer Science and Electrical Engineering, University of Essex, Colchester CO4 3SQ, UK; E-Mail: hhu@essex.ac.uk; 3 School of Information Management and Engineering, Shanghai University of Finance and Economics, Shanghai 200433, China

**Keywords:** fall detection, depth images, Signal-Gauss-Model, Dense spatio-temporal-context

## Abstract

In order to deal with the problem of projection occurring in fall detection with two-dimensional (2D) grey or color images, this paper proposed a robust fall detection method based on spatio-temporal context tracking over three-dimensional (3D) depth images that are captured by the Kinect sensor. In the pre-processing procedure, the parameters of the Single-Gauss-Model (SGM) are estimated and the coefficients of the floor plane equation are extracted from the background images. Once human subject appears in the scene, the silhouette is extracted by SGM and the foreground coefficient of ellipses is used to determine the head position. The dense spatio-temporal context (STC) algorithm is then applied to track the head position and the distance from the head to floor plane is calculated in every following frame of the depth image. When the distance is lower than an adaptive threshold, the centroid height of the human will be used as the second judgment criteria to decide whether a fall incident happened. Lastly, four groups of experiments with different falling directions are performed. Experimental results show that the proposed method can detect fall incidents that occurred in different orientations, and they only need a low computation complexity.

## 1. Introduction

According to a survey from the National Institutes of Health (NIH), there are more than 1.6 million older U.S. adults that suffer fall-relative injuries each year [1]. In 2010, the direct medical cost of fall-related injuries, including fatal and non-fatal falls, in the U.S. was $28.2 billion [2]. In China, news reported that a large contingent of society is growing older at the same time, and 17% of the population will include people older than 60 by 2020. With the aging population growing, the risk of falling and fall-related problems raises consequently. Efficient and robust fall detection systems with low costs will play an important role in reducing fall injuries and their costs. Many falls are linked to older people’s physical condition and home environment. As people get older, their muscles become weak and their balance becomes worse. When older people get up from lying down or sitting, the blood pressure will drop too much, and this increases the chance of falling down. Apart from the fragile bodies of elders, the potential fall hazards (slippery floors, clutter, poor lighting, unstable furniture, obstructed ways, pets, *etc.*) in home environments may also cause people to fall [3,4]. Existing proposed fall detection methods can be divided into three categories: wearable sensor-based, ambient sensor-based, and computer vision-based methods. Wearable sensor-based methods usually rely on accelerometry, posture, or a fusion of the two. In this sort of method, sensors should be attached to the subject’s body, which has high level of obtrusiveness. Ambient sensor-based methods deploy external sensors in the vicinity of the subject. The most available sensor is the pressure sensor since weight or vibrational data can be captured to detect and track the subject. Profiting from the development of computer vision technology, computer vision-based methods have become a promising solution for fall detection systems. Only monocular or multiple cameras are needed in this sort of method to record color images or depth images of a scene. The main advantage is that people do not need to carry specialized sensors, which is makes them more convenient for elders. Fall detection systems usually focus on a single subject. If the scene contains two or more subjects, a segmentation module that will be used to make out each subject can be detected and tracked respectively [5,6]. As most fall events occur in the home environment, the majority of research focuses on this background. 

In order to deal with the problem of projection occurring in fall detection with two-dimensional (2D) grey or color images, this paper presents a robust fall detection method based on spatio-temporal context tracking by analyzing three-dimensional (3D) depth images captured by the Kinect sensor. It is assumed that only one elderly person is in the scene. In the pre-processing procedure, the parameters of the Single Gauss Module (SGM) are estimated and the coefficients of the floor plane equation in the background images are extracted. Once a human subject appears in the scene, the silhouette will be extracted by SGM and the foreground coefficient of the ellipses is used to determine the head position. Then, the dense spatio-temporal context (STC) algorithm is used to track the head position and the distance from the head to the floor plane is calculated in every following frame of the depth image. When the distance is lower than an adaptive threshold, the centroid height of the human will be used as the second judgment to decide whether a fall incident happened. The logical steps of our method are: (1) reference frame is used to extract the human silhouette from the first depth frame and the 3D position of the head is located; (2) the 3D position of head is tracked by using the dense spatio-temporal context learning method; and (3) the distance from the 3D position of head to the floor is calculated in every frame of the depth image. If the height of the head is lower than the threshold, the centroid height of the human will be also be used to judge the threshold to determine a fall. The remainder of this paper is organized as follows. Section 2 reviews the relative works in the area of fall detection; Section 3 describes the proposed fall detection method which depends on the depth data record from the Kinect sensor. Experimental results are presented in Section 4 to show the performances of the proposed approach. Finally, a brief conclusion and thoughts on potential future work are given in Section 5.

## 2. Relative Works

Most existing fall detection methods can be divided into three categories: wearable sensor-based, ambient sensor-based, and computer vision-based methods. We will review the relative work from this perspective. 

### 2.1. Wearable Sensor-Based Methods

In the wearable sensor-based methods, elderly persons need to wear sensors on their body to detect their motion status or determined location. Most wearable sensors (such as the accelerometer and gyroscope) are cheaper than external sensors and are usually easy to operate, though a high level of obtrusiveness is the main disadvantage of wearable sensors [3]. Some systems classify fall and non-fall activities by using the threshold detection method [7,8]. In [7], the fall detection system consisted of an inertial sensor unit, a data logger unit, and a real-time fall detection algorithm. Inertial frame velocity is the main variable to detect falls which can be obtained from transform acceleration and angular velocity. A fixed threshold and an adaptive threshold were applied in the detection algorithm, respectively. By testing 10 young adults and 14 older adults, it was found that the adaptive threshold has a better performance in decreasing the probability of false alarms. However, the threshold’s setting is also a considerable challenge, especially in different fall types [3]. The machine learning classifiers are also widely used in fall detection systems. Özdemir *et al.* presented an automated fall detection system which requires the user to wear motion sensor units (accelerometer, gyroscope, magnetometer/compass) at six different positions [9]. By feature selection and reduction from raw data for each sensor, six machine learning classifiers (k-nearest neighbor (k-NN) classifier, least squares method (LSM), support vector machines (SVM), Bayesian decision making (BDM), dynamic time warping (DTW), and artificial neural networks (ANNs)) were used to classify falls from daily activities. Among all classifiers, the k-NN classifier has shown 99.91% in accuracy, 99.79% in specificity, and 100% in sensitivity. However, it is very inconvenient for elderly people to wear six sensors. Additionally, an external RF connection (ZigBee) is also required to connect with a remote PC. All these elements restrict this sort of approach from obtaining good performance in real-world environments. 

### 2.2. Ambient Sensor-Based Methods

Ambient sensor-based methods usually arrange external sensors around persons’ environments. The common features that are used for person detection and tracking are pressure, vibration, audio, and infrared array, *etc.* [10]. A new fall detection method based on near-field imaging (NFI) was proposed in [11] by Rimminen *et al.* In the experiment, the 19 m^2^ test floor was fully covered with a 9 × 16 matrix of floor sensors to create the NFI. The shape, size, and magnitude of patterns are extracted from the NFI in the feature extraction module. In order to reduce the influence on features from noise and overlap, Bayesian filtering has been used previously. By using a prior two-state Markov chain model, the probability of falling and getting up were used to classify test features. The system obtained a sensitivity of 91% and a specificity of 91% by testing 650 events. However, the system performed moderately when test subjects were ending up sitting or falling onto their knees. Additionally, the sensor matrix will grow exponentially as the test area increases. Zigel *et al.* presented a fall detection system based on floor vibrations and sound sensing [12]. The system contained a training phase and testing phase. In the training phase, 17 features that were extracted from vibration and sound event signals were chosen for classification. Bayes classification algorithm was used in this method. In experiments, this system used a human-mimicking doll to simulate fall events and got a performance with a sensitivity of 97.5% and a specificity of 98.6%. Even though the system is much too sensitive, its precision will decrease if fall events happen farther than 5 m away. In order to obtain more precision, two or more sensors should be set in a large room environment. 

### 2.3. Computer Vision-Based Methods

With the development of computer vision and image processing techniques, computer vision-based methods have become a new branch of fall detection. Compared with other methods, vision-based methods are less intrusive, have higher accuracy, and are more robust. Depth images in particular can easily be collected by the Kinect, which has been widely used as it is robust to light condition changes and detects behavior characteristics well. In [13], Yu *et al.* proposed a novel computer vision-based fall detection system based on a single camera. In order to extract a human body silhouette, the codebook background subtraction technique was used. The ellipse, shape-structure, and position information of silhouettes were used as the extracted features. This system applied one class support vector machine (OCSVM) method to determine the region in the feature space. We must point out that an online scheme was used to make the OCSVM model have a self-adaptive function for new emerging normal postures, and certain rules were added to improve system performance. The experiment from 24 datasets of 12 people, including walking, standing, sitting, lying, and falling activities, showed a fall detection rate of 100% and a false detection rate of 3%. However, the advanced video segmentation algorithms were required in this system to avoid human intervention (to segment the recorded video to clips). This disadvantage restricts the system’s use in practice. In [14], a segmentation module was used as the first step for the video captured by the camera, and a human silhouette was obtained. This system used the ratio of width to height of the silhouette bounding box and the off-diagonal term from the covariance matrix as the features for fall detection since these features have great variation between falls and other activities. In training and the performance module, Hidden Markov model is used for different activities such as falling, keeling, and other activities. 

The problem of projection may occur in fall detection with 2D grey or color images, which makes it difficult to detect falling events that are collinear with the axis of the cameras [15]. As the color camera-based systems contain facial characteristics of the subjects, privacy issues are addressed in many studies at the same time [4]. Researchers usually choose some methods that avoid capturing facial characteristics, such as capturing environment scenes [16] or using depth images [17,18]. In [16], a wearable embedded smart camera is connected around the waist, and the histograms of edge orientations and strengths for each pixel are used as features. An optical flow-based method was employed to classify three activities and good performance was obtained (correct classification rates of falling, sitting, and lying down are 86.66%, 86.8%, and 82.7%, respectively). This system can work effectively both indoors and outdoors, but is highly invasive for subjects. Gasparrini *et al.* has proposed an automatic, privacy-preserving fall detection method for indoor environments based on depth images captured by the Kinect in top-view configuration [17]. Bian *et al.* proposed a robust fall detection approach by using the improved randomized decision tree (RDT) algorithm to extract joints [18]. An *ad-hoc* algorithm is used in a person segment module and all depth blobs of persons are tracked in the tracking module. Once a depth blob is near the floor, a fall will be detected. For the body joints, spine and skeletal information extracted from the Kinect SDK will be inaccurate when the subject falls or lies down on the floor [15,19]. By employing the head joint distance trajectory as an input feature vector, an SVM classifier was used to detect falls or other activities. There were 380 samples in the experiments and good performance was obtained. In order to deal with the problem of projection occurring in fall detection with 2D grey or color images, this paper presents a robust fall detection method based on RGB-D images captured by a Kinect sensor. In the method, a Single-Gauss-Model (SGM) for the background and the spatio-temporal context tracking algorithm are used. 

## 3. The Proposed Method

**Figure 1:**
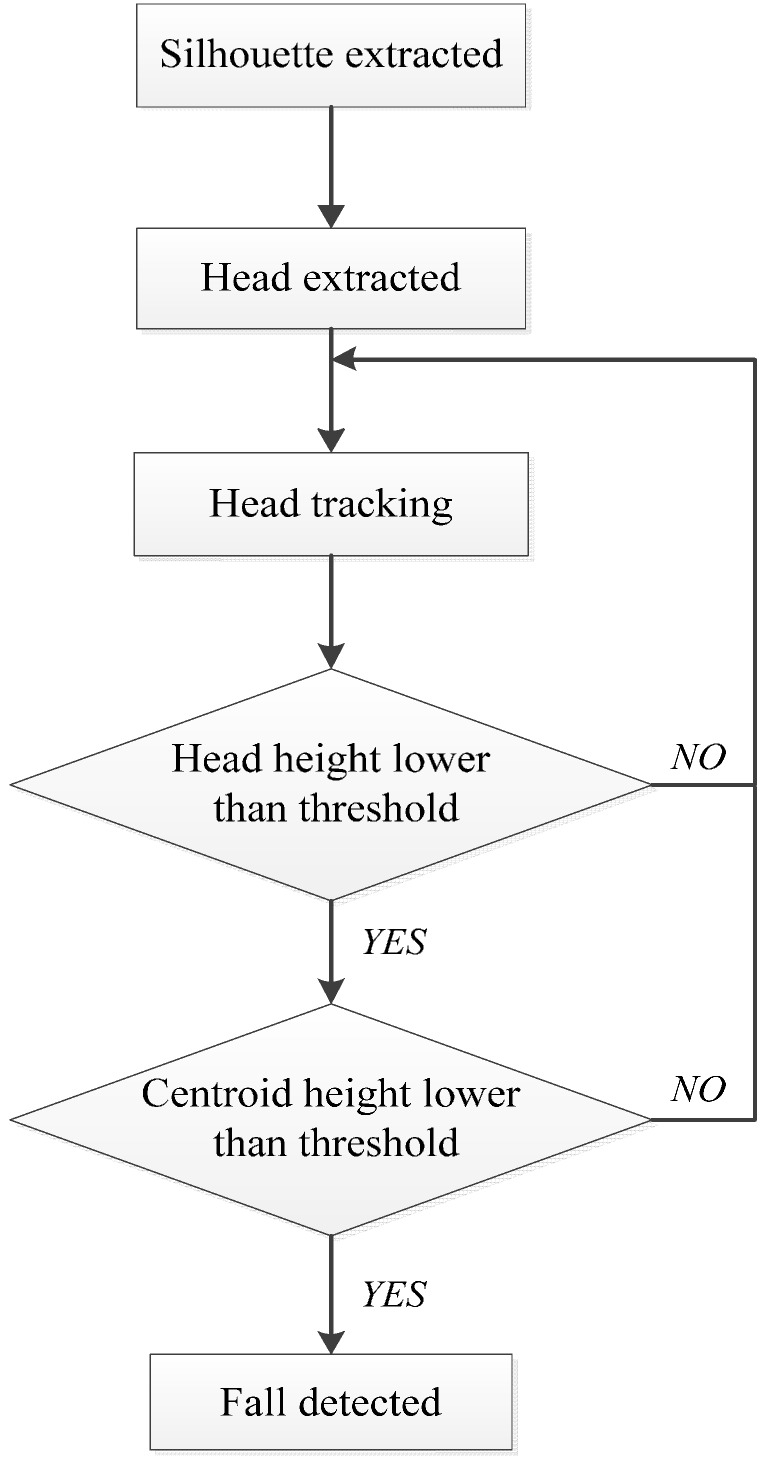
Flow chart of the proposed fall detection method.

In this section, the details of the proposed fall detection method are described. In this method, the head position of the subject and the coefficients of the floor plane equation are obtained first. In the following frames, the tracking algorithm is used to track the head position and the distance from the head to the floor is calculated. When the height of the head is lower than an adaptive threshold, the centroid height will be used as the second judgment to compare with the adaptive threshold. The fall activity may be detected under the condition that both head and centroid heights are lower than the adaptive threshold. The basic flow of our fall detection method is shown in Figure 1. 

### 3.1. Foreground and Centroid Extraction 

During the process of image capture, one Kinect v1 sensor is set in the laboratory hallway with a height of 1.2 m to cover the whole laboratory. As the head and centroid heights are calculated by the distance formula of point to surface, the position of the Kinect sensor is alterable. The RGB images are captured with a 640 × 480 resolution and a 30 fps frame rate, and depth images are captured with a 320 × 240 resolution at the same time. 

**Figure 2:**
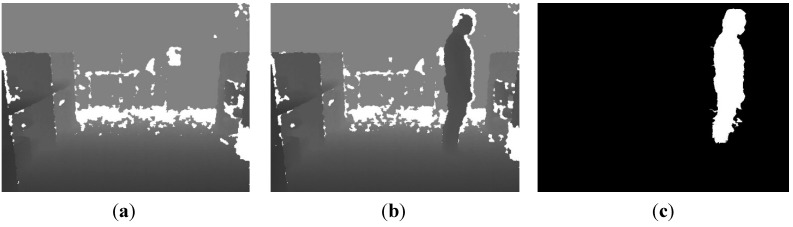
Extraction of human silhouette. (**a**) Background depth frame; (**b**) foreground depth frame; (**c**) extracted human silhouette.

In order to extract the subject’s head position and centroid position, the human silhouette is extracted by the foreground extraction module with the Single Gauss Model (SGM). It is assumed that the measurement noise of the camera has a Gaussian distribution. The SGM contains two parameters which are named mean value *μ* and standard deviation *σ*, as shown in Equations (1) and (2). The parameters can be obtained from *N* depth frames. In our experiments, *N* is 40.

(1) $$\mu \left(i,j\right)=\frac{1}{N}\sum_{n=1}^{N}B\_DF_{n}\left(i,j\right)$$

and

(2) $$\sigma \left(i,j\right)=\sqrt{\frac{1}{N}\sum_{n=1}^{N}\left[B\_DF_{n}\left(i,j\right)-\mu \left(i,j\right)\right]}$$

where *B*_*DF_n_* is the *n*-th background depth frame. It can be proved that the probability within 2.5*σ* is 98.76% in the SGM. Based on this, 2.5*σ* can be used as the threshold to extract the human silhouette with Equation (3), and the extraction result of the human silhouette is shown in Figure 2.

(3) $$Silhouette\left(i,j\right)=\{\begin{array}{c} 0|F\_DF\left(i,j\right)-\mu \left(i,j\right)|<2.5\sigma \left(i,j\right)\\ 1|F\_DF\left(i,j\right)-\mu \left(i,j\right)|>2.5\sigma \left(i,j\right) \end{array}$$

**Figure 3:**
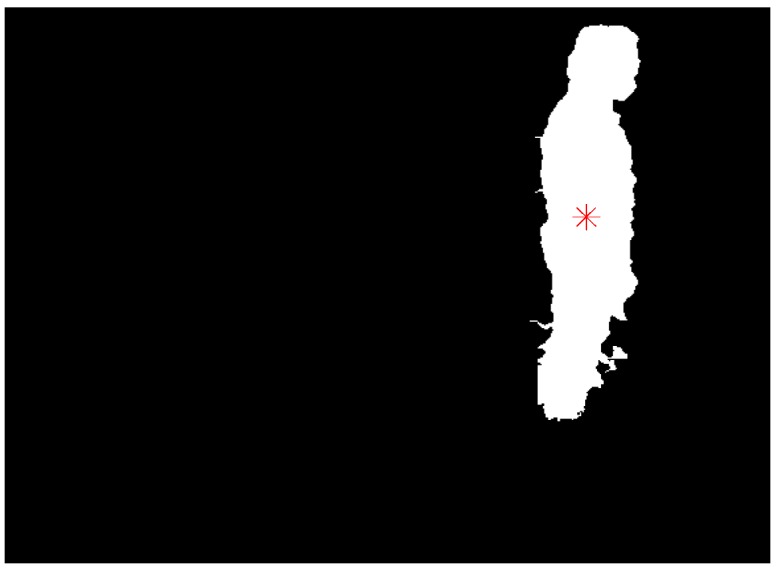
Centroid position of extracted human silhouette.

In order to make the SGM adaptive to changes of scene, it is necessary to update the parameters in real-time as follows:

(4) $$\mu^{k+1}\left(i,j\right)=\{\begin{array}{cc} \mu^{k}\left(i,j\right), & Silhouette\left(i,j\right)=1\\ \left(1-\alpha \right)\mu^{k}\left(i,j\right)+\alpha F\_DF\left(i,j\right), & Silhouette\left(i,j\right)=0 \end{array}$$

and

(5) $$\sigma^{k+1}\left(i,j\right)=\{\begin{array}{cc} \sigma^{k}\left(i,j\right), & Silhouette\left(i,j\right)=1\\ \left(1-\beta \right)\sigma^{k}\left(i,j\right)+\beta {\left[\mu^{k+1}\left(i,j\right)-F\_DF\left(i,j\right)\right]}^{2} & Silhouette\left(i,j\right)=0 \end{array}$$

where *α* and *β* are the learning rates of *μ* and *σ*, respectively. In our experiments *α* and *β* are both 0.05. During the updating process, the parameters of the foreground pixels are kept the same and the background pixels are updated. Through the updating process of the parameters, the potential mutation caused by error parameters of background pixels can be avoided effectively. The distance between the centroid of the silhouette and the floor plane will be used in fall detection. In binary images, the centroid is obtained from Equation (6).

(6) $$\left[\begin{array}{c} C\_x\\ C\_y \end{array}\right]=\left[\begin{array}{c} \frac{\sum xf\left(x,y\right)}{\sum f\left(x,y\right)}\\ \frac{\sum yf\left(x,y\right)}{\sum f\left(x,y\right)} \end{array}\right]$$

where *f*(*x*,*y*) is equal to 1 in the silhouette area and 0 in background area, correspondingly. The centroid position of Figure 2c is marked by a red asterisk in Figure 3. 

### 3.2. Head Position Extraction and Head Tracking

**Figure 4:**
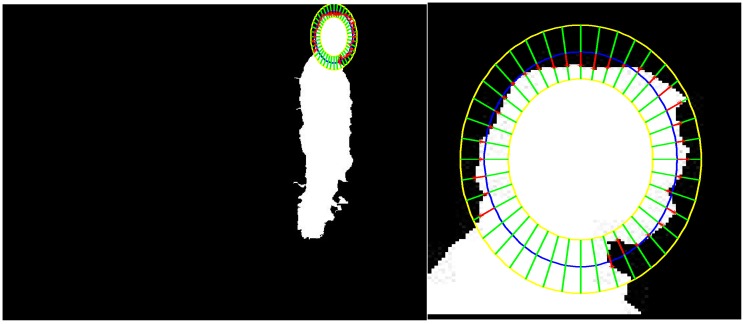
Head extraction result from the human silhouette in Figure 2.

In order to extract the head position, the ellipses are used to find the foreground coefficient by searching for silhouette contour points [20]. In the algorithm, there are three ellipses: the central ellipse is defined by parameters *a* and *b*, the inner ellipse is defined by parameters 3*a*/4 and 3*b*/4, and the outer ellipse is defined by parameters 5*a*/4 and 5*b*/4. In order to compute the foreground coefficient, 40 normal segments of the central ellipse’s contour were used, which started on the inner ellipse and ended on the outer ellipse. It is clear that the foreground coefficient will obtain the maximum value when the central ellipse is fitted with the head edge.

(7) $$C=\frac{1}{N}\sum_{n=1}^{N}\frac{D\left(n\right)-d\left(n\right)}{D\left(n\right)}$$

where *N* is the number of segments, *D* is the half-length of normal segments, and *d* is the distance from the central ellipse point to the silhouette point. The head extraction result of Figure 2c is shown in Figure 4.

Since the head position has been extracted, the dense spatio-temporal context (STC) algorithm [21] is used for tracking proposes. In the algorithm, a Bayesian framework is adopted to formulate the spatio-temporal relationship between the object and its local dense contexts by using a computing confidence map. The maximized confidence map is defined as the new object position. It is assumed that *x* denotes the object position, *o* denotes the object presented in the test frame, and x^*^ denotes the object position in the previous frame. The context feature set is defined as $X^{c}=\left\{c(z)=(I(z),z)|z\in \Omega_{c}(x^{*})\right\}$, where *I*(*z*) is the depth value at position *z* and Ω*_c_*(x^*^) is the neighbor set of x^*^. Then the confidence map is computed by Equation (8).

(8) $$c\left(x\right)=P\left(x|o\right)=\sum_{c\left(z\right)\in X^{c}}P\left(x|c\left(z\right),o\right)P\left(c\left(z\right)|o\right)$$

where *P*(*x*|*c*(*z*),*o*) is the conditional probability that bridges the gap between object position and spatial context, and *P*(*c*(*z*)|*o*) is context prior probability. In the spatial-context model, the conditional probability learns the relativity spatial relations between different pixels as follows:

(9) $$P\left(x|c\left(z\right),o\right)=h^{sc}\left(x-z\right)$$

where *h^sc^* is a function dependent on the distance and direction of object position *x* and spatial context position *z*. Just as the conditional probability is computed from two different characteristics, it can resolve ambiguities effectively when similar objects appear in close proximity. In the context prior model, the context prior probability is computed by the convolution of the depth value of the context position and Gaussian weight shown as Equation (10). Additionally, Fast Fourier Transform (FFT) makes the learning and detection module fast.

(10) $$P\left(c\left(z\right)|o\right)=I\left(z\right)\omega_{\sigma }\left(z-x^{*}\right)$$

### 3.3. Floor Plane Extraction

Fall detection methods are usually based on the distances from head and centroid to floor plane. In [17], the *MaxHeight* depth value and threshold were used to extract the floor plane. The Kinect sensor is placed on the top view configuration specifically, otherwise the floor plane cannot be extracted accurately. In [18], the four coefficients of the floor plane equation are determined by choosing three points from the depth image. However, fewer input points made the system be less robust to noise.

In our algorithm, the least squares method is used to obtain coefficients after choosing the definite floor area. The depth points can defined as (*X_i_*,*Y_i_*,*Z_i_*), where *X_i_*,*Y_i_* and *Z_i_* are depth points’ coordinates in the real world. As the depth points’ coordinates have been determined, the floor plane equation can be described as *AX* + *BY* + *CZ* = 1. Then Equation (11) is satisfied for these depth points.

(11) $$\left[\begin{array}{ccc} X_{1} & Y_{1} & Z_{1}\\ X_{2} & Y_{2} & Z_{2}\\ \vdots & \vdots & \vdots \\ X_{n} & Y_{n} & Z_{n} \end{array}\right]\left[\begin{array}{c} A\\ B\\ C \end{array}\right]=\left[\begin{array}{c} 1\\ 1\\ \vdots \\ 1 \end{array}\right]$$

and the coefficients can be solved by the least squares method shown as

(12) $$\left[\begin{array}{c} A\\ B\\ C \end{array}\right]={\left({\left[\begin{array}{ccc} X_{1} & Y_{1} & Z_{1}\\ X_{2} & Y_{2} & Z_{2}\\ \vdots & \vdots & \vdots \\ X_{n} & Y_{n} & Z_{n} \end{array}\right]}^{T}\left[\begin{array}{ccc} X_{1} & Y_{1} & Z_{1}\\ X_{2} & Y_{2} & Z_{2}\\ \vdots & \vdots & \vdots \\ X_{n} & Y_{n} & Z_{n} \end{array}\right]\right)}^{-1}{\left[\begin{array}{ccc} X_{1} & Y_{1} & Z_{1}\\ X_{2} & Y_{2} & Z_{2}\\ \vdots & \vdots & \vdots \\ X_{n} & Y_{n} & Z_{n} \end{array}\right]}^{T}\left[\begin{array}{c} 1\\ 1\\ \vdots \\ 1 \end{array}\right]$$

After the coefficients of the floor plane equation have been determined, all depth points should be substituted into the floor plane equation. If the subtraction result is smaller than 0.05, the depth points will be defined as the floor plane. The criterion is shown as:

(13) |AX+BY+CZ−1|<0.05

**Figure 5:**
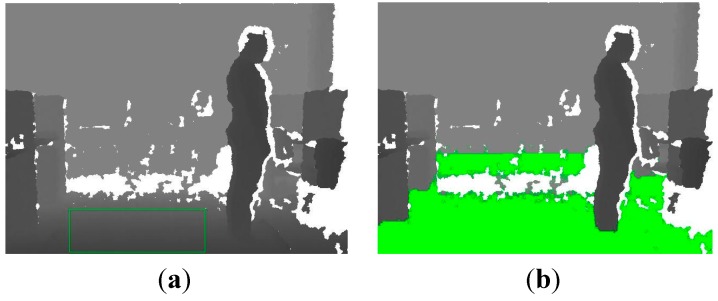
Extracted results of floor plane from depth images. (**a**) Definite domain of floor plane; (**b**) estimated results of floor plane.

As shown in Figure 5, the floor plane has been extracted effectively, and it is robust to illumination and color interference. 

### 3.4. Fall Detection

**Figure 6:**
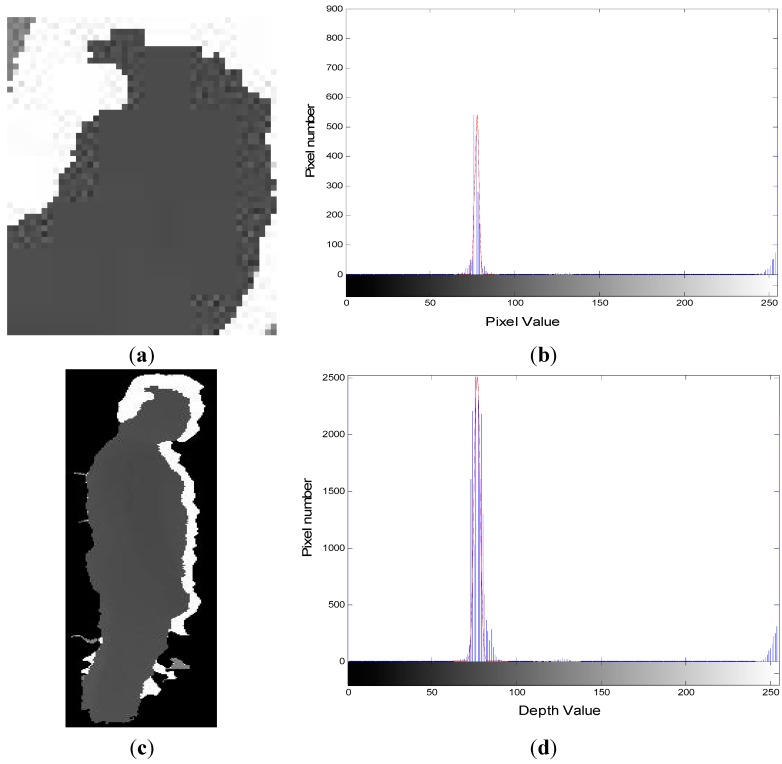
Histogram of head and human in depth images. (**a**) Head depth image; (**b**) histogram of head in depth image; (**c**) human subject depth image; (**d**) histogram of human subject depth image.

The distance between the head and the floor plane is the main criterion in fall detection, and the distance between the silhouette centroid and the floor plane is the second criterion. In order to reduce the distance calculation error caused by noise, the histogram of the depth image is fitted by the Gauss model and the mean value is used as the depth value of the head and centroid of the human, shown as Figure 6. Combined with Section 3.1 and Section 3.2, the 3D position of the head and centroid can be represented as *Head*(*X*,*Y*,*Z*) and *Centroid*(*X*,*Y*,*Z*).

At last, the distance is calculated by Equation (14) from the known floor plane equation.

(14) $$H=\frac{\left|A*X+B*Y+C*Z-1\right|}{\sqrt{A^{2}+B^{2}+C^{2}}}$$

When a person first appears in the scene, the head position will be extracted and tracked, and a sequence of the head height can be calculated which is marked as *H_head*. Then, 0.25*H_head^1^ is used as the threshold, which is suggested by reference [22], where *H_head*^1^ is the first value of the head height that has been calculated. The dynamic threshold made the method adaptive to persons with different heights. If the head height in the tracking process is lower than the threshold, the centroid height *H_centroid* will be used as the second judgment. If head height is not lower than the threshold, the centroid position and centroid height will not be extracted and calculated. This judgment policy can reduce the complexity of the algorithm to a great degree. When the head height and the centroid height are both lower than the threshold at the same time, a fall accident can be determined.

## 4. Experimental Results and Discussion

Most depth image-based fall detection methods were proposed after 2012, and no standard 3D depth imags database is available to evaluate the performance of different methods and mechanisms. In this section, the performance of the proposed method is evaluated by experiments on a dataset with four different orientations. The experiments were performed in the simulated home environment. A Kinect sensor was used for recording the real video sequence. The recorded video sequence is processed by using Matlab2013 on an Intel(R) Core(TM) i5 3.10 GHz CPU and 3 GB RAM. Then the proposed method was applied to fall detection on the dataset. 

**Figure 7:**
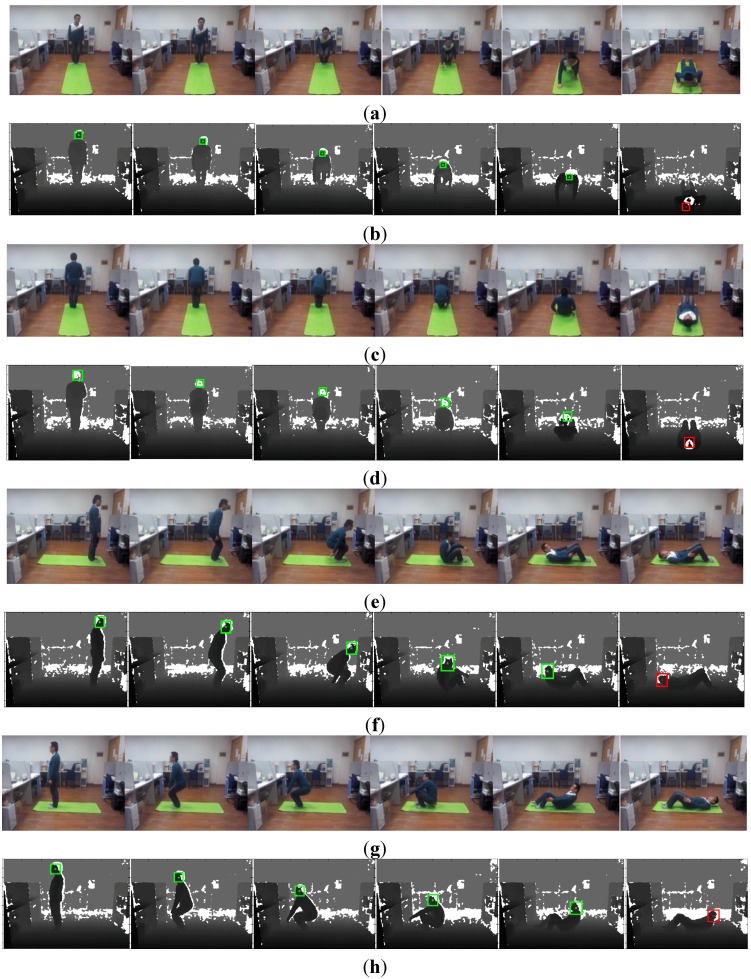
Falling down in different orientations. (**a**) Frames of color images of falling down in the anterior direction; (**b**) frames of depth images of falling down in the anterior direction; (**c**) frames of color images of falling down in the posterior direction; (**d**) frames of depth images of falling down in the posterior direction; (**e**) frames of color images of falling down in left direction; (**f**) frames of depth images of falling down in left direction; (**g**) frames of color images of falling down in right direction; (**h**) frames of depth images of falling down in the right direction.

**Figure 8:**
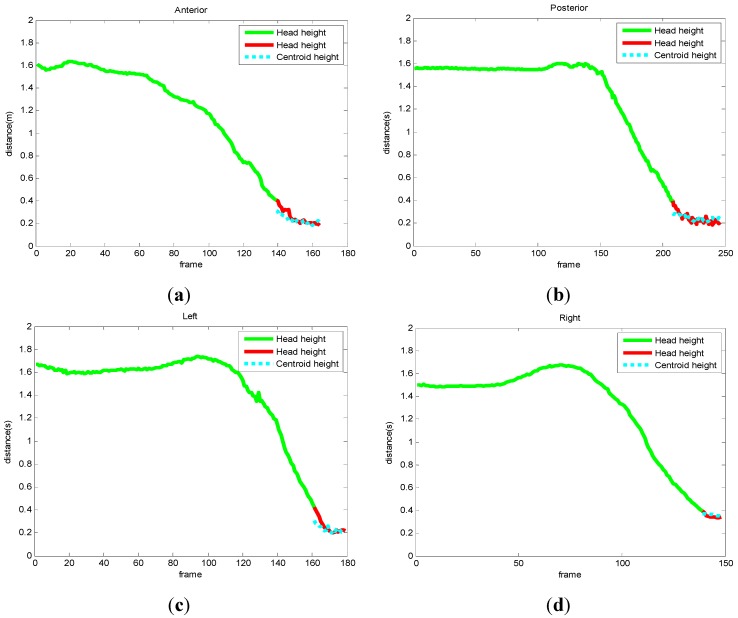
Trajectories of the distance from the head and centroid to the floor in different orientations. (**a**) Falling down from the anterior orientation; (**b**) falling down from the posterior orientation; (**c**) falling down from the left orientation; (**d**) falling down from the right orientation.

Some examples of falls in four different orientations are anterior, posterior, left, and right, as shown in Figure 7. Figure 7a,c,e,g are frames of color images of falling down in four directions, and Figure 7b,d,f,h are frames of depth images of falling down in four directions at the same time. In the first frame of each sequence, the head positions have been extracted by the method shown in Section 3.2 and the threshold can be calculated. The head positions of participants are marked by a green bounding box and the centers of the rectangle are marked by a green asterisk. In the following frames, the head positions are tracked and the distances from the head to the floor plane are computed and compared with the threshold. As long as the distance is lower than the threshold, the distance from the centroid of the human to the floor plane will be used as the second judgment. When the double distances are both lower than the threshold, the participant will be detected as falling. In the last row of each sequence, the fall has been detected and the head position is marked by a red bounding box and a red asterisk. The trajectories of the distances from the head and centroid to the floor plane are shown in Figure 8. Correspondingly, only when the fall has been detected the distance is marked from green to red.

As shown in Figure 7 and Figure 8, the fall incidents have been detected accurately. In general methods, falling is hard to detect when the fall orientation is aligned with the optical axis of the vision sensor [3,4]. In these experiments, we got the right detection results although the orientations are on the optical axis of the Kinect in the anterior and posterior directions. The adaptive threshold enhances this method to fit persons with different heights. One issue with the Kinect is that depth resolution will decrease at large distances, even though is robust to illumination. The accurate distance is only 5 m, which limits its application in large areas [22,23]. As only the depth image is used, there is no need to consider the adjustment between depth images and color images. During the process of head tracking, the sizes of the bounding box change largely as shown in the fourth image of Figure 7. A future version of the tracking algorithm with a different tracking scale will improve the accuracy of fall detection. 

As fall accidents happen in a very short period of time, the fall detection methods are required to obtain fast response abilities. The time for total frames and time per frame of each sequence in our dataset have been measured on our PC and the results are shown in Table 1. It can be concluded that the frame rate of our method is about 43 frames per second, which is faster than the frame rate of common video with 30 frames per second. This is because our method does not need a pre-processing module to handle compact and redundant pixels. 

**Table 1:** Time consumption of the proposed method.

Fall Direction	Time for Total Frames (s)	Time for per Frame (ms)	Frame Number
anterior	3.7241	22.8472	163
posterior	5.4960	22.3415	246
left	4.1075	23.0758	178
right	3.5418	23.9311	148

## 5. Conclusions

In this paper, we have proposed a new robust fast fall detection method which is based on depth images captured from a Kinect sensor. The method only requires the construction of a Single-Gauss-Model and the determination of the coefficients of the floor plane equation. The high speed spatio-temporal context tracking algorithm is applied on the raw depth images to enhance the proposed method and reduce the response time to the fall incident. As the Kinect sensor is cheap, the proposed method can be easily applied in a smart home environment. Most existing fall detection methods depend on posture extraction and classification, and fall and non-fall activities can only be detected in some specific environments by methods of pattern classification. However, all postures in fall activities cannot be included and learned, no matter how much training data has been used. In the proposed method, only the distances from head and centroid to the floor plane are used as judgment, which avoids individual differences effectively. As for practical application in the future, the resolution and scope of the depth image should be improved. The Kinect v2 sensor will be used. Our further research work will be focused on how to solve the problem of the tracking scale as scales are changing during the process of tracking in order to improve the accuracy of head position extraction. Other judgments may also be added in the proposed method so as to enhance its robustness. 

## References

[1] Nihseniorheatlth: About Falls. http://nihseniorhealth.gov/falls/aboutfalls/01.html.

[2] Elizabeth H. The Latest in Falls and Fall Prevention. http://www.cdc.gov/HomeandRecreationalSafety/Falls/fallcost.html.

[3] Delahoz Y.S., Labrador M.A. (2014). Survey on fall detection and fall prevention using wearable and external sensors. Sensors.

[4] Nashwa E.B., Tan Q., Pivot F.C., Anthony L. (2013). Fall detection and prevention for the elderly: A review of trends and challenges. Int. J. Smart Sens. Intell. Syst..

[5] Feng W., Liu R., Zhu M. (2014). Fall detection for elderly person care in a vision-based home surveillance environment using a monocular camera. Signal Image Video Process..

[6] Liao Y.T., Huang C.-L., Hsu S.-C. (2012). Slip and fall event detection using Bayesian Belief Network. Pattern Recognit..

[7] Wu G., Xue S. (2008). Portable preimpact fall detector with inertial sensors. IEEE Trans. Neural Syst. Rehabilit. Eng..

[8] Wang C.-C., Chiang C.-Y., Lin P.-Y., Chou Y.-C., Kuo I.-T., Huang C.-N., Chan C.-T. Development of a fall detecting system for the elderly residents. Proceedings of the 2nd IEEE International Conference on Bioinformatics and Biomedical Engineering (iCBBE2008).

[9] Ozdemir A.T., Barshan B. (2014). Detecting falls with wearable sensors using machine learning techniques. Sensors.

[10] Mubashir M., Shao L., Seed L. (2013). A survey on fall detection: Principles and approaches. Neurocomputing.

[11] Rimminen H., Lindström J., Linnavuo M., Sepponen R. (2010). Detection of falls among the elderly by a floor sensor using the electric near field. IEEE Trans. Inf. Technol. Biomed..

[12] Zigel Y., Litvak D., Gannot I. (2009). A method for automatic fall detection of elderly people using floor vibrations and sound-Proof of concept on human mimicking doll falls. IEEE Trans. Biomed. Eng..

[13] Yu M., Yu Y., Rhuma A., Naqvi S.M., Wang L., Chambers J.A. (2013). An online one class support vector machine-based person-specific fall detection system for monitoring an elderly individual in a room environment. IEEE J. Biomed. Health Inf..

[14] Anderson D., Keller J.M., Skubic M., Chen X., He Z. Recognizing falls from silhouettes. Proceedings of the 28th Annual International Conference of the IEEE Engineering in Medicine and Biology Society.

[15] Planinc R., Kampel M. (2013). Introducing the use of depth data for fall detection. Pers. Ubiquitous Comput..

[16] Ozcan K., Mahabalagiri A.K., Casares M., Velipasalar S. (2013). Automatic Fall Detection and Activity Classification by a Wearable Embedded Smart Camera. IEEE J. Emerg. Sel. Top. Circuits Syst..

[17] Gasparrini S., Cippitelli E., Spinsante S., Gambi E. (2014). A depth-based fall detection system using a Kinect sensor. Sensors.

[18] Bian Z.P., Hou J., Chau L.P., Magnenat-Thalmann N. (2015). Fall detection based on body part tracking using a depth camera. IEEE J. Biomed. Health Inf..

[19] Mastorakis G., Makris D. (2012). Fall detection system using Kinect’s infrared sensor. J. Real-Time Image Process..

[20] Rougier C., Meunier J., St-Arnaud A., Rousseau J. (2013). 3D head tracking for fall detection using a single calibrated camera. Image Vis. Comput..

[21] Zhang K., Zhang L., Liu Q., Zhang D., Yang M.-H. (2014). Fast visual tracking via dense spatio-temporal context learning. Computer Vision–ECCV 2014.

[22] Yang S.-W., Lin S.-K. (2014). Fall detection for multiple pedestrians using depth image processing technique. Comput. Methods Programs Biomed..

[23] Stone E.E., Skubic M. (2015). Fall detection in homes of older adults using the Microsoft Kinect. IEEE J. Biomed. Health Inf..

